# Prognostic value of serum high mobility group box 1 protein and histone H3 levels in patients with disseminated intravascular coagulation: a multicenter prospective cohort study

**DOI:** 10.1186/s12959-022-00390-2

**Published:** 2022-06-13

**Authors:** Hirotaka Mori, Yuki Kataoka, Kayo Harada-Shirado, Noriaki Kawano, Mineji Hayakawa, Yoshinobu Seki, Toshimasa Uchiyama, Kazuma Yamakawa, Hiroyasu Ishikura, Yuhei Irie, Kenji Nishio, Noritaka Yada, Kohji Okamoto, Shingo Yamada, Takayuki Ikezoe

**Affiliations:** 1 Department of Hematology, Fukushima Medical University, 1 Hikarigaoka, Fukushima, 960-1295 Japan; 2 Scientific Research Works Peer Support Group (SRWS-PSG), Osaka, Japan; 3 Department of Internal Medicine, Kyoto Min-Iren Asukai Hospital, 89 Tanaka Asukai-cho, Kyoto, 606-8226 Japan; 4 Section of Clinical Epidemiology, Department of Community Medicine, Kyoto University Graduate School of Medicine, Sakyo-ku, Kyoto, 606-8501 Japan; 5 Department of Healthcare Epidemiology, Kyoto University Graduate School of Medicine/Public Health, Sakyo-ku, Kyoto, 606-8501 Japan; 6 Department of Hematology, Miyazaki Prefectural Miyazaki Hospital, 5-30 Kita Takamatsu-machi, Miyazaki, 880-8510 Japan; 7 Department of Emergency Medicine, Hokkaido University Hospital, Kita-ku, Sapporo, Hokkaido N14W5060-8648 Japan; 8 Department of Hematology, Uonuma Institute of Community Medicine, Niigata University Medical and Dental Hospital, 4132 Urasa, Minamiuonuma-shi, Niigata, 949-7302 Japan; 9 Department of Laboratory Medicine, National Hospital Organization Takasaki General Medical Center, 36 Takamatsu-cho, Takasaki, Gunma 370-0829 Japan; 10 Department of Emergency Medicine, Osaka Medical and Pharmaceutical University, 2-7 Daigaku-machi, Takatsuki, Osaka 569-8686 Japan; 11 Department of Emergency and Critical Care Medicine, Faculty of Medicine, Fukuoka University, 7-45-1 Nanakuma Jonan-ku, Fukuoka, 814-0180 Japan; 12 Department of General Medicine, Nara Medical University, 840 Shijo-cho, Kashihara, Nara 634-8522 Japan; 13 Department of Surgery, Kitakyushu City Yahata Hospital, 2-6-2 Ogura Yahatahigashi-ku, Kitakyushu, Fukuoka 805-8534 Japan; 14 Shino-Test Corporation, R&D Center, Sagamihara, 252-0331 Japan

**Keywords:** Disseminated intravascular coagulation, High mobility group box 1 protein, Histone H3, Prognosis, Discrimination, Calibration, Net reclassification improvement

## Abstract

**Background:**

We compared the prognostic value of serum high mobility group box 1 protein (HMGB1) and histone H3 levels with the International Society on Thrombosis and Haemostasis (ISTH) disseminated intravascular coagulation (DIC) scores for 28-day in-hospital mortality in patients with DIC caused by various underlying diseases.

**Methods:**

We conducted a multicenter prospective cohort study including two hematology departments, four emergency departments, and one general medicine department in Japan, between August 2017 and July 2021. We included patients diagnosed with DIC by the ISTH DIC scoring system.

**Results:**

Overall, 104 patients were included: 50 with hematopoietic disorders, 41 with infections, and 13 with the other diseases. The 28-day in-hospital mortality rate was 21%. The receiver operator characteristic (ROC) curve showed that a DIC score of 6 points, serum HMGB1 level of 8 ng/mL, and serum histone H3 level of 2 ng/mL were the optimal cutoff points. The odds ratios of more than these optimal cutoff points of the DIC score, serum HMGB1, and histone H3 levels were 1.58 (95% confidence interval [CI]: 0.60 to 4.17, *p* = 0.36), 5.47 (95% CI: 1.70 to 17.6, *p* = 0.004), and 9.07 (95% CI: 2.00 to 41.3, *p* = 0.004), respectively. The area under the ROC curve of HMGB1 (0.74, 95% CI: 0.63 to 0.85) was better than that of the ISTH DIC scores (0.55, 95% CI: 0.43 to 0.67, *p* = 0.03), whereas that of histone H3 was not (0.71, 95% CI: 0.60 to 0.82, *p* = 0.07). Calibration and net reclassification plots of HMGB1 identified some high-risk patients, whereas the ISTH DIC scores and histone H3 did not. The category-free net reclassification improvement of HMGB1 was 0.45 (95% CI: 0.01 to 0.90, *p* = 0.04) and that of histone H3 was 0.37 (95% CI: − 0.05 to 0.78, *p* = 0.08).

**Conclusions:**

Serum HMGB1 levels have a prognostic value for mortality in patients with DIC. This finding may help physicians develop treatment strategies.

**Supplementary Information:**

The online version contains supplementary material available at 10.1186/s12959-022-00390-2.

## Background

Disseminated intravascular coagulation (DIC), characterized by systemic hypercoagulation, develops in association with various underlying diseases including sepsis and hematological malignancies [1]. Although the prognosis of patients with DIC has improved, it remains poor. The overall 28-day mortality in patients with DIC decreased mainly due to advances in the fundamental treatment of underlying diseases and anti-DIC treatment strategies during the past 8 years [2]. Nevertheless, a multicenter observational study of 1,895 patients showed that patients with DIC had higher in-hospital mortality than those without DIC (38% vs. 24%, *p* < 0.001) [3]. Therefore, the early identification of patients with severe DIC would facilitate efficacious interventions and risk stratification, which may help tailor effective treatment strategies [4]. Ideally, existing prediction models can be applied to different situations from the development set before developing a new prediction model [5]. However, prognostic prediction in patients with DIC is not well developed. The widely used International Society on Thrombosis and Haemostasis (ISTH) DIC scores [1] were developed for the diagnosis of DIC; however, they did not linearly predict mortality in patients with DIC, although they did in patients with and without DIC [3, 6, 7]. Therefore, a current issue is to examine other prognostic factors.

Recent studies evaluated several molecular biomarkers that were useful for DIC diagnosis and prognostication. These biomarkers include damage associated molecular patterns (DAMPs) such as high mobility group box 1 protein (HMGB1) or histone H3. Pathologically, these proteins are released from damaged or activated cells and play pro-inflammatory and procoagulant roles in DIC development [8]. Clinically, serum HMGB1 and histone H3 levels in patients with sepsis and acute leukemia were elevated in non-survivors [9–13]. In addition, the elevation of serum HMGB1 and histone H3 levels was found in patients with DIC and in patients with organs failure [10, 12, 13]. Regarding laboratory findings, serum HMGB1 and histone H3 levels were correlated with ISTH DIC scores [10, 12, 13]. In particular, unlike platelets or fibrinogen, which comprise the ISTH DIC scoring system, high serum levels of these biomarkers were not relatively affected by underlying disease types such as infectious diseases, hematopoietic disorders, and other diseases [12–14]. Therefore, serum HMGB1 and histone H3 levels in patients with DIC caused by various underlying diseases may be associated with mortality risk. However, to the best of our knowledge, only one study has shown higher serum histone H3 levels in 17 DIC non-survivors than in 17 DIC survivors [15]. Furthermore, the statistical methods did not assess basic prognostic performance, such as discrimination or calibration [16]. Thus, whether serum HMGB1 or histone H3 levels are more useful than DIC scores in predicting the prognosis of patients with DIC caused by various diseases is not investigated.

Accordingly, we conducted this biomarker study to evaluate the prognostic value of serum HMGB1 or histone H3 levels for identifying high-risk patients with DIC compared with the ISTH DIC scores.

## Methods

### Study design and setting

This multicenter prospective cohort study used the Japanese Society on Thrombosis and Hemostasis (JSTH) committee registry with coagulopathy data planned a priori. This study was registered in the University Hospital Medical Information Network Clinical Trial Registry in August 2017 (UMIN-CTR ID: UMIN000032972). Registry data were collected from August 2017 to July 2021 from two hematology centers (Fukushima Medical University Hospital and Miyazaki Prefectural Miyazaki Hospital), four emergency centers (Hokkaido University Hospital, Nara Medical University Hospital, Osaka General Medical Center, and Fukuoka University Hospital), and one clinical laboratory department in an acute hospital (Takasaki General Medical Center) in Japan, for a total of seven centers. All patients or their families were provided written informed consent, which was approved by each institution’s ethics committee, before collecting patient characteristics and blood samples. Our statistical analysis referred to the Standards for Reporting Diagnostic Accuracy (STARD) statement [17] in Supplementary Table S1 (Additional file 1) because our study was relevant to medical tests, and the statement explains that most STARD items would still apply to evaluate prognosis.

### Patients

We consecutively included ISTH DIC patients [18] (Supplementary Table S2; Additional file 2) with the following inclusion criteria: (1) aged ≥ 16 years and (2) requiring hospitalization or urgent care for the treatment of underlying disease of DIC [14]. The exclusion criteria were as follows: (1) coagulation disorders due to obstetric and gynecological diseases, (2) blood transfusion performed before the assessment of the inclusion criteria, or (3) treatment of underlying diseases started before blood sample collection.

### Data collection

We developed a clinical research form and collected the following data: age, sex, underlying diseases, and underlying disease types using the JSTH classification including hematopoietic disorder, infectious diseases, and the others classified as basic type [14]; laboratory tests including platelet counts, D-dimer, prothrombin time international normalized ratio (PT-INR), fibrinogen, HMGB1, and histone H3; administration of recombinant human soluble thrombomodulin (rhTM); duration of administration of rhTM; administration of antithrombin; and 28-day in-hospital mortality. Samples for laboratory tests were collected before treatment. The underlying diseases of all the patients were treated according to the attending physician’s decisions. The platelet count, PT-INR, and fibrinogen levels were analyzed using an automated counting device at each institution. Serum samples were stored at − 80 °C after centrifugation and sent to the assay companies for other coagulation tests. Serum D-dimer levels were measured using the LPIA-GENESIS D-dimer at the LSI Medience (Tokyo, Japan). Serum HMGB1 and histone H3 levels were measured using an enzyme-linked immunosorbent assay (ELISA) at the Shino-Test Corporation (Kanagawa, Japan) based on previously reported methods [19, 20]. The attending physician chose which anticoagulants, antithrombin, or DIC scoring systems to use or not. The attending physicians were blinded to the serum HMGB1 or histone H3 levels. The measurers of serum HMGB1 and histone H3 levels were blinded to the patients’ clinical information.

Compliance with the data form was monitored. Additionally, we interviewed the physicians completing the data form for missing information. We conducted these post hoc interviews within three months after the data from laboratory companies were obtained.

### Outcome measurement

The main outcome was 28-day in-hospital mortality at the time of study entry. Patients discharged after treatment completion or those who remained in the hospital for more than 28 days were considered alive. We confirmed the survival of patients transferred to other departments within 28 days.

### Statistical analysis

First, we summarized patients’ characteristics using median and interquartile range for continuous variables and percentage for categorical variables for all patients, survivors, and non-survivors. Continuous and categorical variables were compared between survivors and non-survivors using the Mann–Whitney–Wilcoxon and chi-square tests, respectively.

Second, we evaluated the associations between mortality and the ISTH DIC scores, serum HMGB1, and histone H3 levels. After calculating the percentage of mortality at each point on the ISTH DIC scores, we calculated the odds ratios of the ISTH DIC scores. Before calculating the odds ratio of serum HMGB1 and histone H3 levels, we graphically checked whether the relationship between serum HMGB1 and histone H3 levels as continuous variables and mortality was linear in the log of the odds ratio for mortality with a smoothing curve using a locally weighted least squares (Lowess) regression. Next, we described the receiver operating characteristic (ROC) curve and explored the optimal cutoff point of the ISTH DIC scores, serum HMGB1, and histone H3 levels from ROC. Subsequently, we calculated the odds ratio of the ISTH DIC scores, serum HMGB1, and histone H3 levels as dichotomized variables with optimal cutoff points.

Third, we compared predictive abilities of the ISTH DIC scores, serum HMGB1, and histone H3 levels for mortality. Discrimination was compared using the area under the ROC curve (AUC) with 95% confidence interval (CI). Calibration was graphically assessed using a calibration plot and numerically evaluated using the Hosmer–Lemeshow (HL) test. The diagonal line in the calibration plot was regarded as reference for good calibration. Finally, we evaluated category-free net reclassification improvement (NRI), which measures the improvement in discrimination [21]. Additionally, we calculated the category additive and absolute NRIs [16]. Usually, a category is created using cutoff points of the same predicted probabilities between two models [22]. However, the range of predicted probability of the ISTH DIC scores was considered rather narrow to create risk categories compared with serum HMGB1 and histone H3 levels because the ISTH DIC scores could have only four values from 5 to 8 points; serum HMGB1 and histone H3 levels were infinite continuous variables. Therefore, instead of using the predicted probability, we created a risk category using the optimal cutoff points of the DIC scores, serum HMGB1, and histone H3 levels. The low- or high-risk groups for mortality were defined as those below or above the cutoff points of the variables, respectively. The *p*-value in the AUC was the null hypothesis that the AUC of the DIC score was equal to that of HMGB1 and histone H3. The *p*-value in the HL test was the null hypothesis that there was no difference between the observed and predicted mortality rates in divided groups. The *p*-value in the category-free NRI was the hypothesis that there was no change in the predicted probabilities between the ISTH DIC scores and serum HMGB1 or histone H3 levels. The *p*-value in the category NRI was based on the hypothesis that the cutoff point of serum HMGB1 or histone H3 levels does not improve the reclassification of patients classified by the cutoff point of the DIC scores to high or low risk. Statistical significance was set at *p*-value < 0.05.

A complete case analysis was performed. Therefore, we did not estimate the sample size a priori and used all available samples. Data were analyzed using STATA software, V. 15 (StataCorp., College Station, TX, USA) and R software, V 0.4.1.2 (http://www.r-project.org).

## Results

### Patient characteristics

Table 1 presents the patient characteristics.

**Table 1:** Patient characteristics

	**Survivor**	**Non-survivor**	*p*-value*
	***n* = 82**	***n* = 22**	
Demographics			
Age (years), median (IQR)	66 (56 to 76)	70 (62 to 79)	0.16
Male, n (%)	49 (60)	14 (64)	0.74
Underlying disease types			
Hematopoietic disorders, n (%)	38 (46)	12 (55)	0.49
Infectious diseases, n (%)	36 (44)	5 (23)	0.07
The others, n (%)	8 (10)	5 (23)	0.10
Laboratory tests			
Platelet (× 10^9^/L), median (IQR)	40 (22 to 60)	18.5 (12 to 40)	0.03
D-dimer (µg/mL), median (IQR)	16.3 (9.6 to 43.2)	16.2 (11 to 111.2)	0.27
PT-INR, median (IQR)	1.40 (1.13 to 1.55)	1.47 (1.27 to 1.70)	0.22
Fibrinogen (g/L), median (IQR)	2.9 (1.2 to 4.1)	1.6 (1.2 to 3.0)	0.12
HMGB1^a^ (ng/mL), median (IQR)	6.8 (3.1 to 13.6)	16.3 (8.5 to 66.9)	< 0.001
Histone H3^a^ (ng/mL), median (IQR)	2.1 (0.4 to 7.8)	4.8 (2.4 to 31.6)	0.002
DIC score (point), median (IQR)	5 (5 to 6)	5 (5 to 6)	0.40
5 points, n (%)	57 (70)	13 (59)	0.35
6 points, n (%)	18 (22)	7 (32)	0.34
7 points, n (%)	7 (9)	2 (9)	0.93
8 points, n (%)	NA	NA	NA
Anti-DIC agents			
rhTM, n (%)	50 (61)	18 (82)	0.07
Antithrombin, n (%)	18 (22)	8 (36)	0.17

*PT-INR* prothrombin time-international normalized ratio, *HMGB1* high mobility group box-1 protein, *DIC* disseminated intravascular coagulation, *rhTM* recombinant human soluble thrombomodulin, *IQR* interquartile range, *NA* not applicable

^*^ *p*-value is the hypothesis that the proportion or median of the two groups between survivors and non-survivors is the same

^a^ HMGB1 and histone H3 data were missing for the same patient

In total, 104 patients from seven hospitals were eligible for this study. Among the 104 patients, 22 (21%) died. Only one patient had missing information on HMGB1 and histone H3. Mortality did not differ among the ISTH DIC scores of 5, 6, and 7 points. Serum HMGB1 levels in survivors and non-survivors were 6.8 ng/mL (95% CI: 3.1 to 13.6) and 16.3 ng/mL (95% CI: 8.5 to 66.9, *p* < 0.001). Serum histone H3 levels in survivors and non-survivors were 2.1 ng/mL (95% CI: 0.4 to 7.8) and 4.8 ng/mL (95% CI: 2.4 to 31.6, *p* = 0.002). Supplementary Table S3 (Additional file 3) and Supplementary Fig. S1 (Additional file 4) showed serum HMGB1 and histone H3 levels between survivors and non-survivors by underlying disease types separately. No differences were observed in the use of anti-DIC agents. The underlying diseases were summarized in Supplementary Table S4 (Additional file 5).

### Association between mortality and DIC score, serum HMGB1, and histone H3 levels

The 28-day mortality rates at 5, 6, and 7 points were 19% (13/70), 28% (7/25), and 22% (2/9), respectively. The odds ratio for mortality was 1.28 (95% CI: 0.64 to 2.57, *p* = 0.48) per 1 point rise in the DIC score. The association between serum HMGB1 and histone H3 levels and mortality was almost linear in the logit model as shown in Supplementary Fig. S2 (Additional file 6). The odds ratio for mortality was 1.02 (95% CI: 1.00 to 1.03, *p* = 0.02) per 1 ng/mL rise in serum HMGB1 levels and 1.02 (95% CI: 1.00 to 1.04, *p* = 0.04) per 1 ng/mL rise in serum histone H3 levels.

ROC curve showed that 6 points in the DIC score, 8 ng/mL in the serum HMGB1 level, and 2 ng/mL in the serum histone H3 level were the optimal cutoff points. The odds ratios of the DIC score, serum HMGB1, and histone H3 levels as dichotomized variables with the optimal cutoff points were 1.58 (95% CI: 0.60 to 4.17, *P* = 0.36), 5.47 (95% CI: 1.70 to 17.6, *p* = 0.004), and 9.07 (95% CI: 2.00 to 41.3, *p* = 0.004), respectively. Table 2 summarizes the associations between mortality and DIC scores, serum HMGB1, and histone H3 levels.

**Table 2:** Association between mortality and DIC scores, HMGB1, and histone H3

Variables	Odds ratio (95% CI)	*p*-value*
DIC score (point)	1.28 (0.64 to 2.57)	0.48
HMGB1 (ng/mL)	1.02 (1.00 to 1.03)	0.02
Histone H3 (ng/mL)	1.02 (1.00 to 1.04)	0.04
Optimal cutoff point		
DIC score ≥ 6 point	1.58 (0.60 to 4.17)	0.36
HMGB1 ≥ 8 (ng/mL)	5.47 (1.70 to 17.6)	0.004
Histone H3 ≥ 2 (ng/mL)	9.07 (2.00 to 41.3)	0.004

*DIC* disseminated intravascular coagulation, *HMGB1* high mobility group box-1 protein

^*^ *p*-value is the null hypothesis that the odds ratio is equal to one

### Comparison of prediction abilities for mortality between DIC score vs. HMGB1 and histone H3

Table 3 shows the prognostic predictive abilities of the DIC scores and serum HMGB1 and histone H3 levels.

**Table 3:** Comparison of discrimination, calibration, and NRI between DIC scores vs. HMGB1 and histone H3

	**DIC scores**	**HMGB1**	*p*-value*	**Histone H3**	*p*-value*
Discrimination					
AUC, (95% CI)	0.55 (0.43 to 0.67)	0.74 (0.63 to 0.85)	0.03	0.71 (0.60 to 0.82)	0.07
Calibration					
HL test, *P*-value	0.93	0.49	NA	0.10	NA
Category-free NRI					
Overall NRI, (95% CI)		0.45 (0.01 to 0.90)	0.04	0.37 (− 0.05 to 0.78)	0.08
Event NRI		− 0.18		− 0.36	
Non-event NRI		0.63		0.73	

*DIC* disseminated intravascular coagulation, *HMGB1* high mobility group box-1 protein, *AUC* area under the receiver operating characteristic curve, *HL* Hosmer–Lemeshow, *NRI* net reclassification improvement, *NA* not applicable

^*^ *p*-value is the null hypothesis that each prediction index of the DIC scoring system is equal to that of HMG1 and histone H3

The AUC of the DIC scores, serum HMGB1, and histone H3 levels was 0.55 (95% CI: 0.43 to 0.67), 0.74 (95% CI: 0.63 to 0.85), 0.71 (95% CI: 0.60 to 0.82), respectively (Supplementary Fig. S3; Additional file 7). The differences in the AUC between serum HMGB1 levels and the DIC scores and serum histone H3 levels and the DIC scores were 0.19 (*p* = 0.03) and 0. 16 (*p* = 0.07), respectively. Supplementary Table S5 showed the AUC of platelet counts, D-dimer, PT-INR, fibrinogen, DIC scores, serum HMGB1, and histone H3 levels (Additional file 8).

**Fig. 1:**
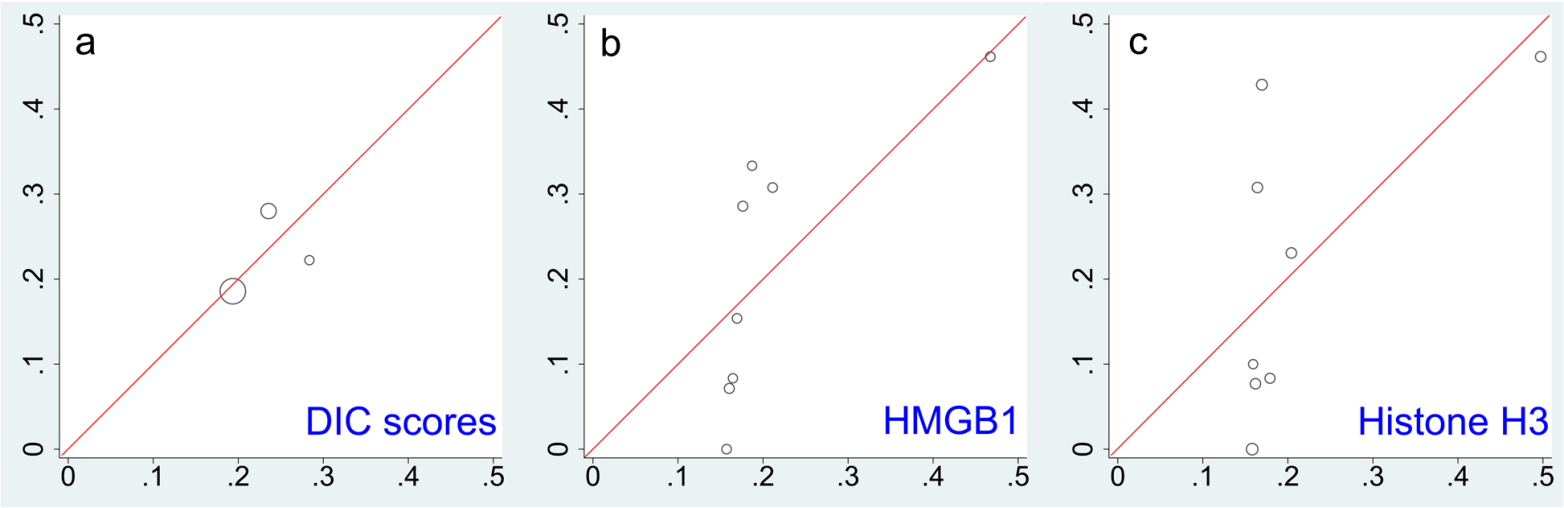
Calibration plot. **a** Calibration plot of DIC score. **b** Calibration plot of HMGB1. **c** Calibration plot of histone H3. The x-axis represents the predicted proportion of the DIC scores (**a**), HMGB1 (**b**), and histone H3 (**c**). The y-axis represents the observed proportions. The diagonal line represents the reference for good calibration. The size of the circles indicates the number of patients. DIC, disseminated intravascular coagulation; HMGB1, high mobility group box-1 protein

The *p*-values in the HL test for the DIC scores, serum HMGB1, and histone H3 levels were 0.93, 0.49, and 0.10, respectively. The DIC scores had a good calibration plot in the range of approximately 20%, and serum HMGB1 levels were in the range of approximately 45% (Fig. 1a, b). The serum histone H3 levels did not show a good calibration plot (Fig. 1c).

**Fig. 2:**
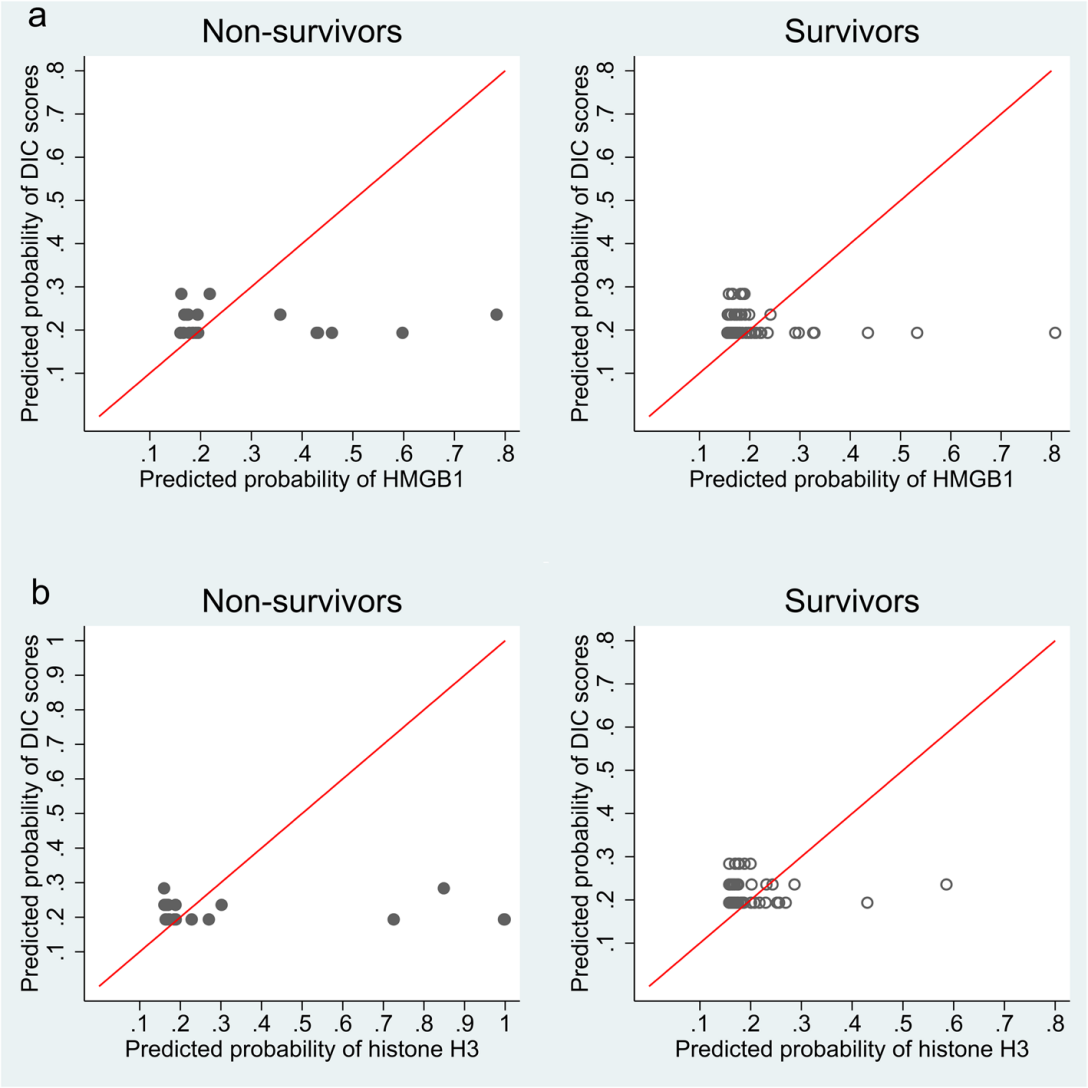
Category-free reclassification plot. **a** Category-free reclassification plot between the predicted probability of the DIC scores and serum HMGB1 levels. **b** Category-free reclassification plot between the predicted probability of the DIC scores and serum histone H3 levels. The diagonal line represents the reference for no change. The closed circle indicates a non-survivor. The open circle represents a survivor. DIC, disseminated intravascular coagulation; HMGB1, high mobility group box-1 protein

The overall category-free NRI by serum HMGB1 levels against the DIC score was 0.45 (95% CI: 0.01 to 0.90, *p* = 0.04). The overall category-free NRI by serum histone H3 levels against the DIC score was 0.37 (95% CI: − 0.05 to 0.78, *p* = 0.08). The event-and non-event-category-free NRIs are listed in Table 3. Figure 2 shows the category-free reclassification plot between the predicted probability of DIC scores and that of serum HMGB1 and histone H3 levels.

The overall category additive NRI by serum HMGB1 levels at the cutoff point of 8 ng/mL vs. DIC score at the cutoff point of 6 points was 26 (95% CI: − 14 to 67, *p* = 0.10), and absolute NRI was − 3% (Supplementary Table S6; Additional file 9). The overall category additive NRI by serum histone H3 levels at the cutoff point of 2 ng/mL vs. DIC score at the cutoff point of 6 points was 28 (95% CI: − 19 to 66, *p* = 0.07), and absolute NRI was − 7% (Supplementary Table S7; Additional file 10). Supplementary Fig. S4 showed the category-free reclassification plot between the predicted probability of DIC scores and that of serum HMGB1 by underlying disease types separately (Additional file 11). Supplementary Fig. S5 showed the category-free reclassification plot between the predicted probability of DIC scores and that of serum histone H3 by underlying disease types separately (Additional file 12).

## Discussion

This multicenter prospective cohort study in patients with DIC caused by various underlying diseases showed that serum HMGB1 levels had significantly higher discrimination and category-free NRI with high non-event category-free NRI compared with the ISTH DIC scores. Additionally, serum HMGB1 levels dotted part of the calibration and reclassification plots in a high probability that the ISTH DIC scores could not dot. The additive category NRI by HMGB1 was 26, with a high net reclassification in non-survivors, which was not significant. Serum histone H3 levels had the same discrimination as serum HMGB1 but did not improve discrimination and NRI compared with the ISTH DIC scores.

Serum HMGB1 levels in patients with DIC may be more useful than the ISTH DIC scores in selecting patients with a high mortality risk. The discrimination of serum HMGB1 levels was better than that of the ISTH DIC scores, which was almost no better than chance. The category-free NRI explains the difference in discrimination. Serum HMGB1 levels assigned lower predicted probabilities to survivors among patients with high predicted probabilities by the ISTH DIC scores. As calibration and reclassification plots showed, serum HMGB1 levels also identified a high-risk group that the ISTH DIC scores did not. Additionally, the additive category NRI with high net reclassification in non-survivors suggested that more than 8 ng/mL serum HMGB1 levels better classified non-survivors with an ISTH DIC score of 5 points as the low-risk group to the high-risk group. The clinical value of discrimination, calibration, and NRI results is specific to clinical practice in a field [16]. Regarding DIC, identification of high-risk patients with DIC may increase survival benefits of anticoagulants [23]. Accordingly, considering the proposed DIC treatment strategy and our results, physicians may intensify the underlying disease treatments of patients identified as high-risk by HMGB1 in combination with anticoagulants.

The clinical usefulness of serum histone H3 levels may be limited. Although histone H3 showed a high odds ratio, its calibration was worse than that of HMGB1. Additionally, the discrimination and overall category-free NRI did not differ significantly from the ISTH DIC scores. The lower absolute NRI by histone H3 compared with HMGB1 suggested a higher risk of misclassification. Therefore, clinicians could not use histone H3 as a high-risk biomarker of DIC. However, histones, including histone H3, contribute to the development of DIC by interacting with prothrombin and increasing the efficiency of thrombin production by factor X; they may be targeted for treating systemic coagulation activation [24]. Thus, the prognostic value of histone H3 in patients with DIC should be investigated in future.

The strength of our study is that it prospectively evaluated the prognostic value of serum HMGB1 and histone H3 levels simultaneously in patients with DIC in various settings and underlying diseases. Generally, one of the purposes of a prospective and multicenter prognostic prediction study including patients with various diseases is to warrant generalizability, such as reproducibility and transportability, which are as important as accuracy [25]. In our study, patients with DIC had various underlying diseases, and patient characteristics showed a good balance between survivors and non-survivors. Considering an earlier study measuring serum histone H3 levels in 34 patients with DIC [15] and the difficult research feasibility that serum HMGB1 and histone H3 levels are currently measured only for research purposes, the number of 104 patients with DIC in this study may be large. Thus, our results may be applied to, or compared with, future research.

This study has several limitations. First, our sample size was approximately 100 patients and relatively small in terms of the development of clinical prediction rules. According to a recent methodological study, the sample size based on the 20% mortality rate in this study to develop a clinical prediction rule was 250 patients [26]. Therefore, these results may be optimistic and do not create risk categories, including low, intermediate, and high-risk groups [27]. Second, our study did not reach a clear conclusion as to whether the prognostic value of serum HMGB1 and histone H3 levels depending on the underlying disease types and DIC types such as enhanced-fibrinolysis and suppressed-fibrinolysis [28]. Third, we excluded the obstetric and gynecological diseases from this study because the dynamics of basic coagulation markers such as platelet count, D-dimer, PT-INR, and fibrinogen in obstetric and gynecologic diseases are clearly different compared to other diseases, classifying these diseases as a distinct coagulation disease group [29]. Fourth, we did not evaluate the prognostic value of other DIC scoring systems such as the Japanese Ministry of Health and Welfare (JMHW) [30], Japanese Association for Acute Medicine (JAAM) [31], and JSTH DIC scores [14]. Our registry lacked the plan to collect JMHW and JAAM score data in the same patient and the available data was small. Likewise, we did not have enough sample size to evaluate JSTH DIC scores because the criteria of JSTH DIC scoring system varies among underlying disease types [14]. In addition, we did not evaluate other coagulation markers such as antithrombin, thrombin-antithrombin complex, soluble fibrin, and prothrombin fragment 1 + 2. Because these markers are included in JSTH DIC scoring system [14], it is methodologically reasonable to compare the value of these markers and JSTH DIC scores with that of HMGB1 and histone H3. Moreover, we did not investigate the prognostic value of not only other DAMPs such as adenosine triphosphate, mitochondrial formyl peptides, and mitochondrial DNA [32] but also other biomarkers such as plasmin-α2 plasmin inhibitor complex, plasminogen activator inhibitor-1, α2 plasmin inhibitor, protein C, and degradation products of cross-linked fibrin by leukocyte elastase [14]. Therefore, this study may show only the usefulness of serum HMGB1 levels compared with that of the ISTH DIC scores. Fifth, because of these limitations, we did not evaluate the prognostic value of serum HMGB1 levels in addition to the existing DIC scoring systems for mortality in patients with DIC. When the prognostic value of new markers is suggested, adding the markers to existing models is proposed as a reasonable way to evaluate the markers as potential predictors [33]. Sixth, regarding the treatment strategy, because our study design was not intended to evaluate the treatments effect based on serum HMGB1 and histone H3 levels, the usefulness of these markers in making treatment decisions remains unclear.

There were some limitations to these results. Although the category-free NRI was significant, the plots were dense with low risk. Most marginal changes in low predicted risk may not contribute to changes in clinical management [34]. In addition, calibration of HMGB1 at low risk was poor, partly because of the small sample size. An accurate calibration evaluation requires an adequate sample size [35].

Future studies should validate the values of HMGB1 and histone H3 in the large number of patients with established DIC scoring systems, various underlying disease types including obstetric and gynecological diseases, DIC types with enhanced-fibrinolysis and suppressed-fibrinolysis, other DAMPs, and coagulation biomarkers. The updating or development of DIC scoring systems in combination with serum HMGB1 levels in patients with DIC is also needed. To clarify whether the elevation of serum HMGB1 and histone H3 levels is a phenomenon related to underlying diseases or high-risk mortality patients, a future study with enough sample size to conduct factor analysis is also necessary. In addition, whether these biomarkers and DIC scoring systems are useful in determining treatment strategies in various fields should be investigated.

## Conclusions

Clinicians may have more plausible reasons for using serum HMGB1 levels as a high-risk biomarker in patients with DIC. Researchers should update the prediction models for mortality in patients with DIC by combining HMGB1 with existing prediction models or other predictors.

## Supplementary Information

**Additional file 1: Supplementary Table S1. **STARD statement checklist.

**Additional file 2: Supplementary Table S2. **ISTH DIC scoring system.

**Additional file 3: Supplementary Table S3. **Serum HMGB1 and histone H3 levels between survivors and non-survivors by underlying disease types.

**Additional file 4: Supplementary Fig. S1. **Serum HMGB1 levels between survivors and non-survivors by underlying disease types. **a** Serum HMGB1 levels between survivors and non-survivors by underlying disease types. **b** Serum histone H3 levels between survivors and non-survivors by underlying disease types. HMGB1, high mobility group box-1 protein.

**Additional file 5: Supplementary Table S4. **Underlying diseases of DIC.

**Additional file 6: Supplementary Fig. S2. **Locally weighted scatterplot smoothing curve. **a** Relationship between serum HMGB1 levels and mortality. **b** Relationship between serum histone H3 levels and mortality. HMGB1, high mobility group box-1 protein.

**Additional file 7: Supplementary Fig. S3. **Receiver operating characteristic curve of DIC score, HMGB1, and histone H3. The blue line with circles, red line with diamonds, and yellow line with triangles represent the ISTH DIC scores, HMGB1, and histone H3, respectively. The diagonal line indicates reference that discrimination is no better than chance. DIC, disseminated intravascular coagulation; HMGB1, high mobility group box-1 protein; AUC, area under the receiver operating characteristic curve; ISTH, International Society on Thrombosis and Haemostasis.

**Additional file 8: Supplementary Table S5. **AUC of platelet counts, D-dimer, PT-INR, fibrinogen, ISTH DIC scores, HMGB1 and histone H3.

**Additional file 9: Supplementary Table S6. **Category reclassification by HMGB1.

**Additional file 10: Supplementary Table S7. **Category reclassification by histone H3.

**Additional file 11: Supplementary Fig S4. **Category-free reclassification plot between the predicted probability of the DIC scores and serum HMGB1 levels by underlying disease types. The diagonal line represents the reference for no change. DIC, disseminated intravascular coagulation; HMGB1, high mobility group box-1 protein.

**Additional file 12: Supplementary Fig S5. **Category-free reclassification plot between the predicted probability of the DIC scores and serum histone H3 levels by underlying disease types. The diagonal line represents the reference for no change. DIC, disseminated intravascular coagulation.

## Data Availability

Data are available upon reasonable request to the corresponding author.

## Abbreviations

DIC: Disseminated intravascular coagulation
DAMPs: Damage-associated molecular patterns
HMGB1: High mobility group box 1 protein
ISTH: International Society on Thrombosis and Haemostasis
STARD: The Standards for Reporting Diagnostic Accuracy
JSTH: Japanese Society on Thrombosis and Haemostasis
PT-INR: Prothrombin time international normalized ratio
rhTM: Recombinant human soluble thrombomodulin
Lowess: Locally weighted least squares
ROC: Receiver operating characteristic curve
AUC: Area under the receiver operating characteristic curve
HL: Hosmer–Lemeshow
NRI: Net reclassification improvement
