# Correction: Prognostic value of plasma high mobility group box 1 protein and histone H3 levels in patients with disseminated intravascular coagulation: a multicenter prospective cohort study

**DOI:** 10.1186/s12959-022-00406-x

**Published:** 2022-08-31

**Authors:** Hirotaka Mori, Yuki Kataoka, Kayo Harada-Shirado, Noriaki Kawano, Mineji Hayakawa, Yoshinobu Seki, Toshimasa Uchiyama, Kazuma Yamakawa, Hiroyasu Ishikura, Yuhei Irie, Kenji Nishio, Noritaka Yada, Kohji Okamoto, Shingo Yamada, Takayuki Ikezoe

**Affiliations:** 1 Department of Hematology, Fukushima Medical University, 1 Hikarigaoka, Fukushima, 960-1295 Japan; 2 Scientific Research Works Peer Support Group (SRWS-PSG), Osaka, Japan; 3 Department of Internal Medicine, Kyoto Min-Iren Asukai Hospital, 89 Tanaka Asukai-cho, Kyoto, 606-8226 Japan; 4 Section of Clinical Epidemiology, Department of Community Medicine, Kyoto University Graduate School of Medicine, Sakyo-ku, Kyoto, 606-8501 Japan; 5 Department of Healthcare Epidemiology, Kyoto University Graduate School of Medicine/ Public Health, Sakyo-ku, Kyoto, 606-8501 Japan; 6 Department of Hematology, Miyazaki Prefectural Miyazaki Hospital, 5-30 Kita Takamatsu-machi, Miyazaki, 880-8510 Japan; 7 Department of Emergency Medicine, Hokkaido University Hospital, Kita-ku, Sapporo, Hokkaido N14W5060-8648 Japan; 8 Department of Hematology, Uonuma Institute of Community Medicine, Niigata University Medical and Dental Hospital, 4132 Urasa, Minamiuonuma-shi, Niigata, 949-7302 Japan; 9 Department of Laboratory Medicine, National Hospital Organization Takasaki General Medical Center, 36 Takamatsu-cho, Takasaki, Gunma 370-0829 Japan; 10 Department of Emergency Medicine, Osaka Medical and Pharmaceutical University, 2-7 Daigaku-machi, Takatsuki, Osaka, 569-8686 Japan; 11 Department of Emergency and Critical Care Medicine, Faculty of Medicine, Fukuoka University, 7-45-1 Nanakuma Jonan-ku, Fukuoka, 814-0180 Japan; 12 Department of General Medicine, Nara Medical University, 840 Shijo-cho, Kashihara, Nara, 634-8522 Japan; 13 Department of Surgery, Kitakyushu City Yahata Hospital, 2-6-2 Ogura Yahatahigashi-ku, Kitakyushu, Fukuoka, 805-8534 Japan; 14 Shino-Test Corporation, R&D Center, Sagamihara, 252-0331 Japan

**Correction: Thrombosis J 20, 33 (2022)**

**https://doi.org/10.1186/s12959-022-00390-2**

Following publication of the original article [1], it was reported that the term ‘plasma’ should have been used in place of ‘serum’ in the title, abstract, methods, results, discussion, conclusions, and supplementaly information, and prior researches in reference number 11, 12, 13, and 15. It was also reported that the term ‘plasma or serum’ should have been used in place of ‘serum’ in a prior research in reference number nine. The original article [1] has been updated.
