# Supplementary material for: Prognostic value of serum high mobility group box 1 protein and histone H3 levels in patients with disseminated intravascular coagulation: a multicenter prospective cohort study
Source: Thromb J. 2022 Jun 13;20:33. doi: 10.1186/s12959-022-00390-2 (PMC9190102; doi:10.1186/s12959-022-00390-2)
Supplement: Supplementary file 2 — Additional file 2: Supplementary Table S2. ISTH DIC scoring system. [file 12959_2022_390_MOESM2_ESM.docx]

| **Supplementary Table S2. ISTH DIC scoring system** | |
| --- | --- |
| **Items** | **Points** |
| Platelet counts (×10⁹/L) |  |
| ≥ 100 | 0 |
| 50 to < 100 | 1 |
| < 50 | 2 |
| D-dimer (μg/mL) |  |
| < 0.4 | 0 |
| 0.4 to < 4 | 2 |
| ≥ 4 | 3 |
| PT-INR |  |
| < 1.4 | 0 |
| 1.4 to < 2.3 | 1 |
| ≥ 2.3 | 2 |
| Fibrinogen level (g/L) |  |
| ≥ 1 | 0 |
| < 1 | 1 |

DIC was diagnosed using the ISTH DIC scoring system [18].

ISTH, International Society on Thrombosis and Haemostasis; PT-INR, prothrombin time-international normalized ratio; DIC, disseminated intravascular coagulation

Patients with sum scores of ≥ 5 points are diagnosed as DIC.
