# Supplementary material for: Prognostic value of serum high mobility group box 1 protein and histone H3 levels in patients with disseminated intravascular coagulation: a multicenter prospective cohort study
Source: Thromb J. 2022 Jun 13;20:33. doi: 10.1186/s12959-022-00390-2 (PMC9190102; doi:10.1186/s12959-022-00390-2)
Supplement: Supplementary file 3 — Additional file 3: Supplementary Table S3. Serum HMGB1 and histone H3 levels between survivors and non-survivors by underlying disease types. [file 12959_2022_390_MOESM3_ESM.docx]

| **Supplementary Table S3. Serum HMGB1 and histone H3 levels between survivors and non-survivors by underlying disease types.** | | | |
| --- | --- | --- | --- |
|  | **Survivors** | **Non-survivors** | ***p*-value*** |
| Underlying disease types |  |  |  |
| Hematopoietic disorders, n (%) | 38 (46) | 12 (55) | 0.49 |
| Infectious diseases, n (%) | 36 (44) | 5 (23) | 0.07 |
| The others, n (%) | 8 (10) | 5 (23) | 0.10 |
| DIC scores |  |  |  |
| Hematopoietic disorders, median (IQR) | 5 (5 to 6) | 5 (5 to 6) | 0.36 |
| Infectious diseases, median (IQR) | 5 (5 to 6) | 6 (5 to 6) | 0.19 |
| The others, median (IQR) | 5.5 (5 to 6) | 5 (5 to 5) | 0.27 |
| HMGB1 |  |  |  |
| Hematopoietic disorders, median (IQR) | 7.9 (3.1 to 22.8) | 21.55 (12.05 to 88.55) | 0.006 |
| Infectious diseases, median (IQR) | 5.75 (3.1 to 10.55) | 12.9 (6.4-16.9) | 0.11 |
| The others, median (IQR) | 6.35 (2.75 to 10.0) | 8.5 (5.7 to 15.6) | 0.31 |
| Histone H3 |  |  |  |
| Hematopoietic disorders, median (IQR) | 2.1 (0.2 to7.8) | 10 (4.3 to 98.1) | <0.001 |
| Infectious diseases, median (IQR) | 1.95 (0.95 to 7.8) | 2.0 (1 to 2.1) | 0.78 |
| The others, median (IQR) | 2.8 (0.1 to 20.35) | 5.1 (3.3 to 31.6) | 0.34 |

DIC, disseminated intravascular coagulation; HMGB1, high mobility group box-1 protein

**p*-value is the hypothesis that the median of the two groups between survivors and non-survivors is the same.
