# Supplementary material for: Prognostic value of serum high mobility group box 1 protein and histone H3 levels in patients with disseminated intravascular coagulation: a multicenter prospective cohort study
Source: Thromb J. 2022 Jun 13;20:33. doi: 10.1186/s12959-022-00390-2 (PMC9190102; doi:10.1186/s12959-022-00390-2)
Supplement: Supplementary file 5 — Additional file 5: Supplementary Table S4. Underlying diseases of DIC. [file 12959_2022_390_MOESM5_ESM.docx]

| **Supplementary Table S4. Underlying diseases of DIC** | |
| --- | --- |
|  | **Overall** |
| **Underlying diseases** | **n = 104** |
| Infections |  |
| Sepsis, n (%) | 17 (16) |
| Other severe infections, n (%) | 24 (23) |
| Hematopoietic malignancies |  |
| Acute leukemia, n (%) | 35 (34) |
| Malignant lymphoma, n (%) | 10 (9) |
| Other hematopoietic malignancies, n (%) | 5 (5) |
| Solid cancers, n (%) | 4 (4) |
| Tissue damage, n (%) | 4 (4) |
| Vascular-related diseases |  |
| Thoracic or abdominal aortic aneurysms, n (%) | 2 (2) |
| Other vascular-related diseases, n (%) | 3 (3) |

DIC, disseminated intravascular coagulation
