# Supplementary figures and images for: Prognostic value of serum high mobility group box 1 protein and histone H3 levels in patients with disseminated intravascular coagulation: a multicenter prospective cohort study
Source: Thromb J. 2022 Jun 13;20:33. doi: 10.1186/s12959-022-00390-2 (PMC9190102; doi:10.1186/s12959-022-00390-2)

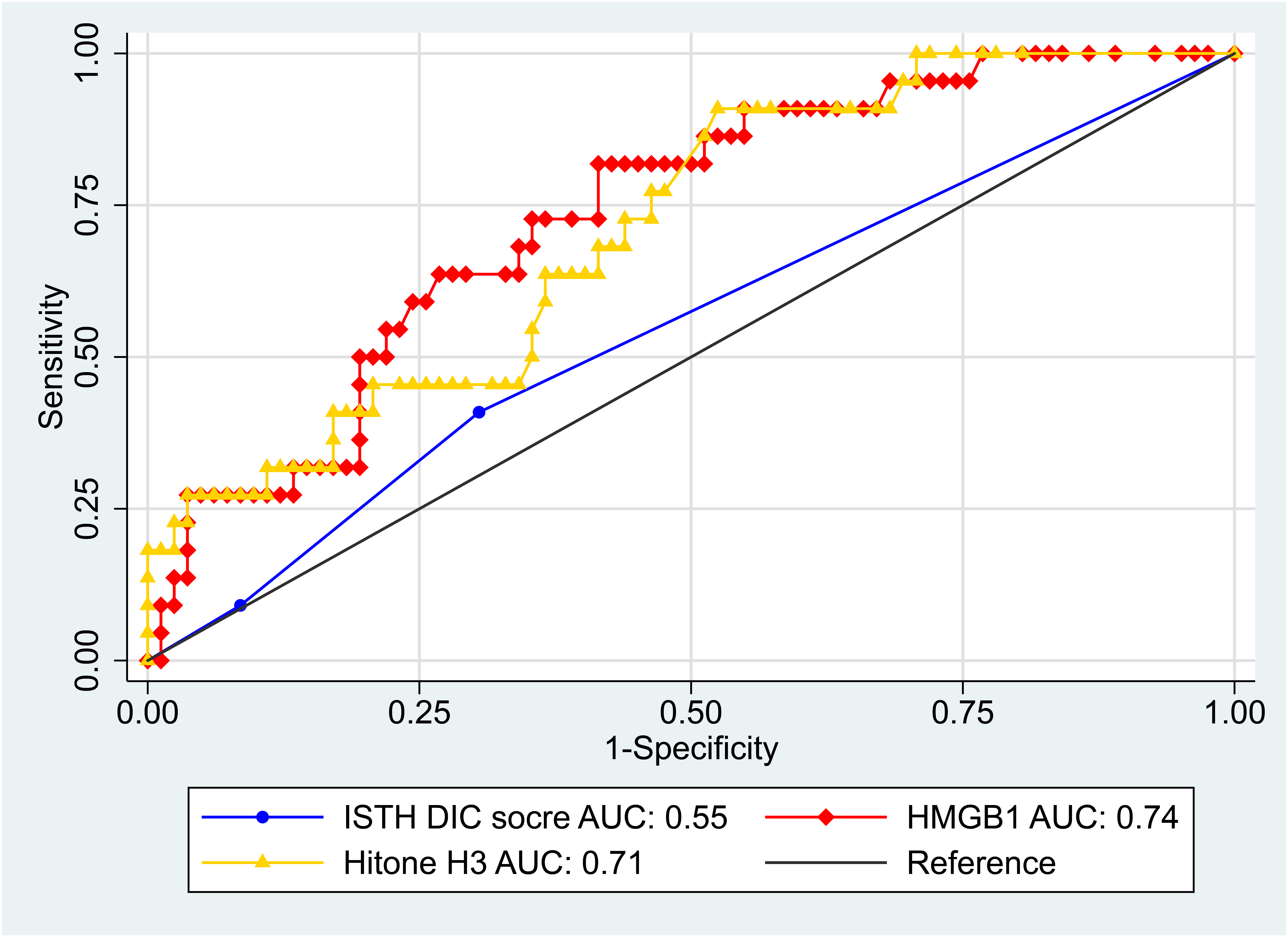

Supplement: Supplementary file 7 — Additional file 7: Supplementary Fig. S3. Receiver operating characteristic curve of DIC score, HMGB1, and histone H3. The blue line with circles, red line with diamonds, and yellow line with triangles represent the ISTH DIC scores, HMGB1, and histone H3, respectively. The diagonal line indicates reference that discrimination is no better than chance. DIC, disseminated intravascular coagulation; HMGB1, high mobility group box-1 protein; AUC, area under the receiver operating characteristic curve; ISTH, International Society on Thrombosis and Haemostasis. [file 12959_2022_390_MOESM7_ESM.tif]

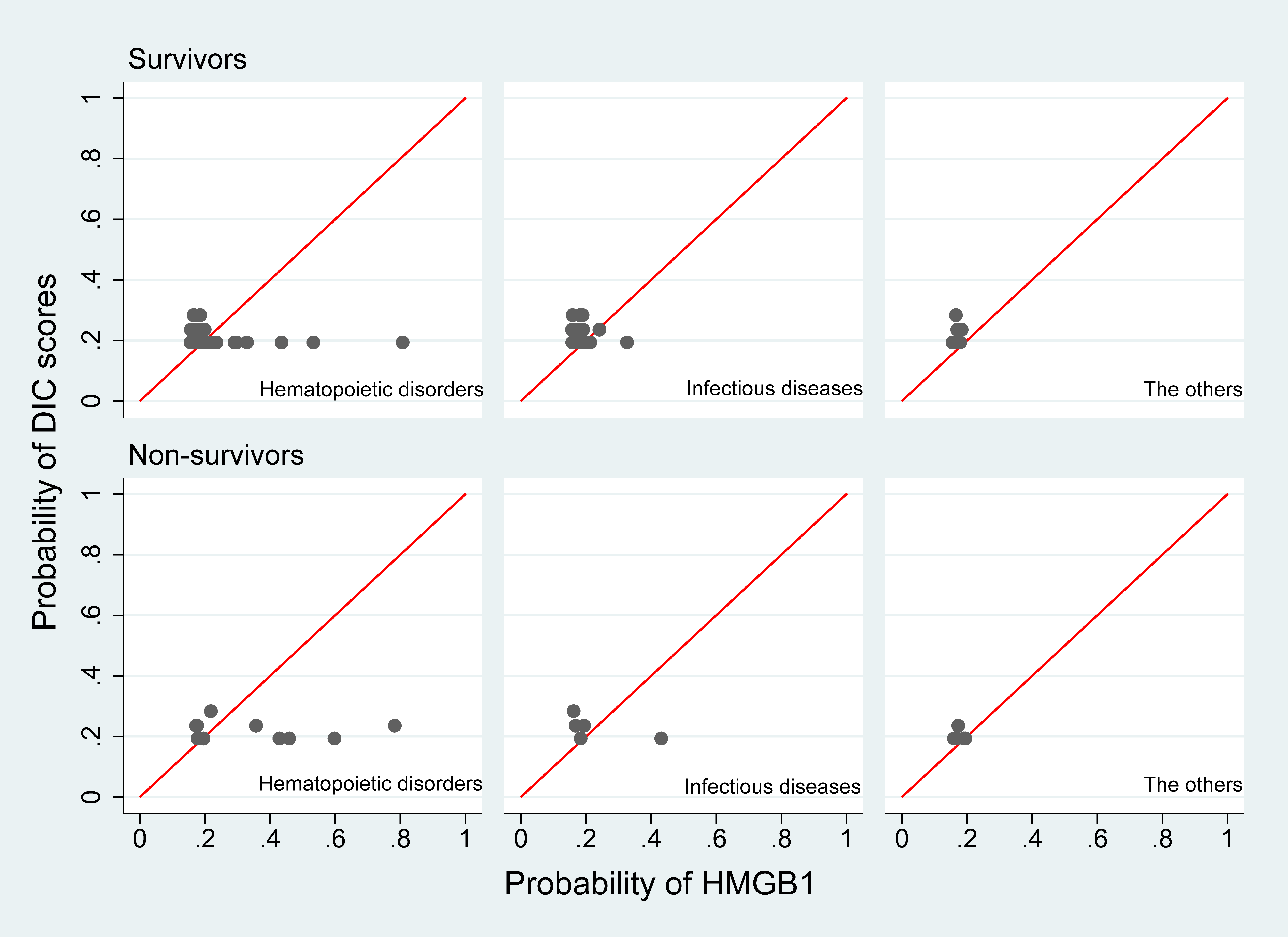

Supplement: Supplementary file 11 — Additional file 11: Supplementary Fig S4. Category-free reclassification plot between the predicted probability of the DIC scores and serum HMGB1 levels by underlying disease types. The diagonal line represents the reference for no change. DIC, disseminated intravascular coagulation; HMGB1, high mobility group box-1 protein. [file 12959_2022_390_MOESM11_ESM.tif]

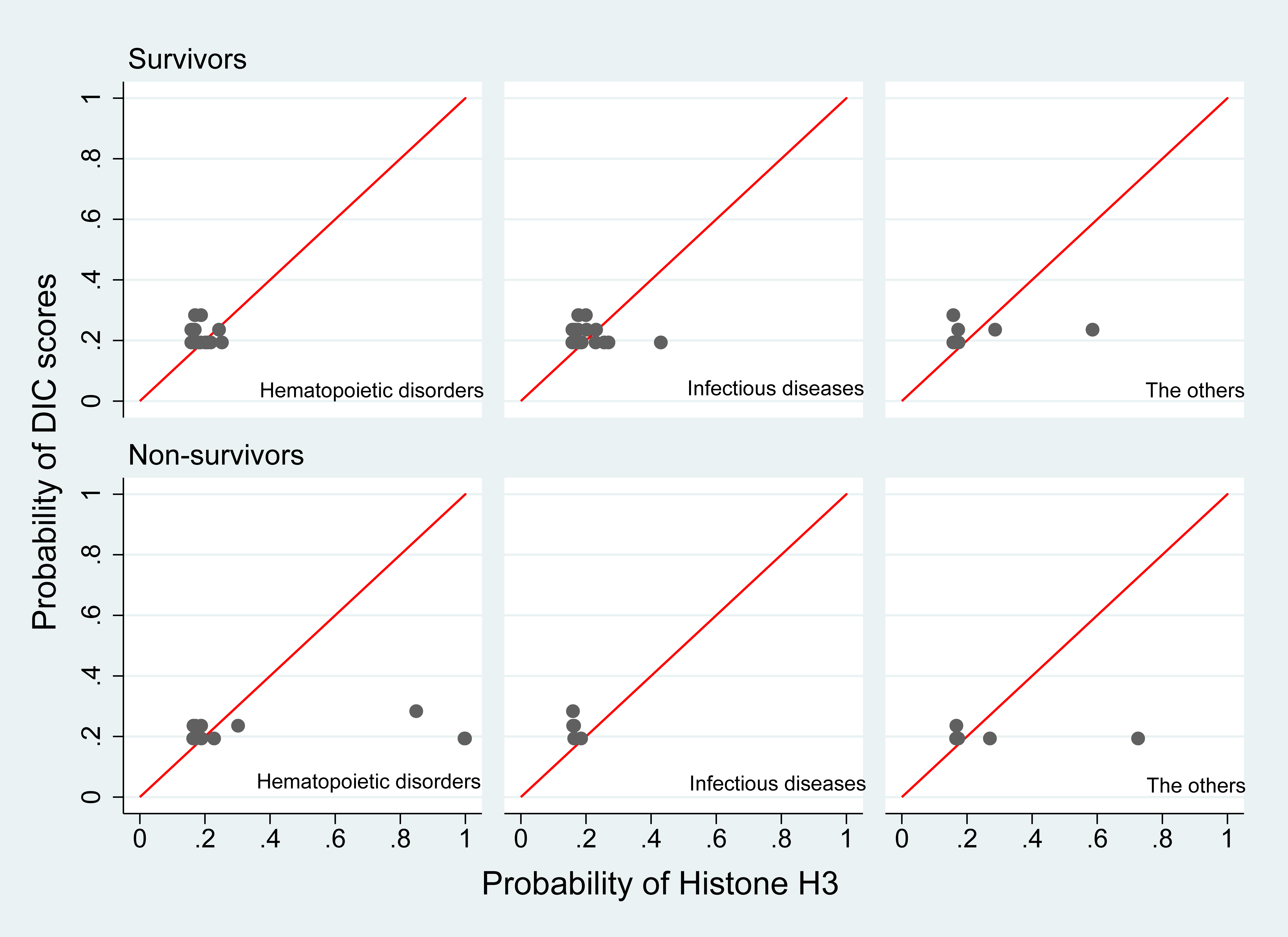

Supplement: Supplementary file 12 — Additional file 12: Supplementary Fig S5. Category-free reclassification plot between the predicted probability of the DIC scores and serum histone H3 levels by underlying disease types. The diagonal line represents the reference for no change. DIC, disseminated intravascular coagulation. [file 12959_2022_390_MOESM12_ESM.tif]
