# Supplementary material for: Prognostic value of serum high mobility group box 1 protein and histone H3 levels in patients with disseminated intravascular coagulation: a multicenter prospective cohort study
Source: Thromb J. 2022 Jun 13;20:33. doi: 10.1186/s12959-022-00390-2 (PMC9190102; doi:10.1186/s12959-022-00390-2)
Supplement: Supplementary file 8 — Additional file 8: Supplementary Table S5. AUC of platelet counts, D-dimer, PT-INR, fibrinogen, ISTH DIC scores, HMGB1 and histone H3. [file 12959_2022_390_MOESM8_ESM.docx]

| **Supplementary Table S5. AUC of platelet counts, D-dimer, PT-INR, fibrinogen, ISTH DIC scores, HMGB1 and histone H3** | |
| --- | --- |
| **Biomarkers or scores** | **AUC (95% CI)** |
| Platelet counts | 0.35 (0.20 to 0.48) |
| D-dimer | 0.58 (0.43 to 0.73) |
| PT-INR | 0.59 (0.47 to 0.70) |
| Fibrinogen | 0.39 (0.26 to 0.52) |
| DIC scores | 0.55 (0.43 to 0.67) |
| HMGB1 | 0.74 (0.63 to 0.85) |
| Histone H3 | 0.71 (0.60 to 0.82) |

AUC, area under the receiver operating characteristic curve; PT-INR, prothrombin time international normalized ratio; DIC, disseminated intravascular coagulation; HMGB1, high mobility group box-1 protein
