# Supplementary material for: Prognostic value of serum high mobility group box 1 protein and histone H3 levels in patients with disseminated intravascular coagulation: a multicenter prospective cohort study
Source: Thromb J. 2022 Jun 13;20:33. doi: 10.1186/s12959-022-00390-2 (PMC9190102; doi:10.1186/s12959-022-00390-2)
Supplement: Supplementary file 9 — Additional file 9: Supplementary Table S6. Category reclassification by HMGB1. [file 12959_2022_390_MOESM9_ESM.docx]

| **Supplementary Table S6. Category reclassification by HMGB1** | | |
| --- | --- | --- |
| **DIC score** | **HMGB1** |  |
|  | **< 8 ng/mL** | **≥ 8 ng/mL** |
| In 22 non-survivors |  |  |
| 5 points | 2 | 11 |
| ≥ 6 points | 2 | 7 |
|  |  |  |
| In 82 survivors |  |  |
| 5 points | 31 | 26 |
| ≥ 6 points | 14 | 11 |

The additive NRI was 0.26 (95% confidence interval: −0.14 to 0.67, *p* = 0.10) and the absolute NRI was −3%.

DIC, disseminated intravascular coagulation; HMGB1, high mobility group box-1 protein; NRI, net reclassification index
